# Predictive value of IGF2BP2 for endometrial cancer recurrence: a multicenter study

**DOI:** 10.3389/fonc.2026.1733447

**Published:** 2026-02-06

**Authors:** Jie Xiong, Peng Jiang, Xue Bai, Yuan Tu, Chenfan Tian, Chunxia Gong, Yu Gong, Lamei Hou, Limei Zhao, Rui Yuan

**Affiliations:** 1 Department of Gynecology, Liangjiang Hospital of Chongqing Medical University, Chongqing Liangjiang New Area People’s Hospital, Chongqing, China; 2 Department of Gynecology, The First Affiliated Hospital of Chongqing Medical University, Chongqing, China; 3 Department of Obstetrics and Gynecology, Women and Children’s Hospital of Chongqing Medical University, Chongqing, China; 4 Department of Gynecology, Suining Central Hospital, Suining, Sichuan, China; 5 Department of Gynecology, Fengdu People’s Hospital, Chongqing, China

**Keywords:** classical parameters, endometrial cancer, IGF2BP2, nomogram model, recurrence

## Abstract

**Background:**

Predictive value of IGF2BP2 in combination with clinicopathological parameters for postoperative recurrence in endometrial cancer (EC): development and validation of a prognostic model.

**Methods:**

This multicenter study retrospectively enrolled patients with endometrial cancer who underwent standard surgical treatment between January 2016 and January 2023. The cohort included 545 patients from the First Affiliated Hospital of Chongqing Medical University (training set) and 315 patients from two independent centers—Liangjiang Hospital of Chongqing Medical University and Women and Children’s Hospital of Chongqing Medical University (validation set). Univariate and multivariate Cox regression analyses were conducted to identify independent prognostic factors associated with recurrence-free survival (RFS), followed by the development of a nomogram-based prediction model. Model discrimination was evaluated using the area under the receiver operating characteristic curve (AUC), and calibration curves were used to assess the agreement between predicted and observed outcomes. Risk stratification was performed according to nomogram-derived scores, and the clinical applicability of the model was further validated through Kaplan-Meier survival analysis.

**Results:**

Multivariate Cox regression analysis identified International Federation of Gynecology and Obstetrics(FIGO) stage (p=0.001), depth of myometrial invasion (p=0.004), histologic type (p=0.001), CA125 level (p=0.001), p53 status (p=0.013), lymphovascular space invasion (p=0.007), and IGF2BP2 expression (p<0.001) as independent prognostic factors for RFS in endometrial cancer patients. The integrated prediction model incorporating these factors demonstrated excellent performance in predicting 1-, 3-, and 5-year RFS, with significantly superior discriminative ability (AUC = 0.884) compared to single-parameter models.

**Conclusion:**

The nomogram integrating IGF2BP2 with clinicopathological parameters demonstrates robust accuracy for predicting recurrence-free survival in endometrial cancer patients. This tool provides a quantitative risk stratification framework that could potentially contribute to prognostic assessment, though its clinical implementation awaits validation in prospective studies.

## Introduction

EC ranks among the most common malignancies of the female reproductive system, with a lifetime risk of 3.1% (1). Notably, it has surpassed ovarian cancer as the leading cause of gynecological cancer-related mortality in the United States (2). Despite advances in understanding its pathogenesis, risk factors, molecular classification, and treatment options, the global incidence of EC continues to rise (3). Over the past two decades, incidence rates have increased 20-fold across all age groups (4). Among women over 50 years of age with an intact uterus, EC is the second most frequently diagnosed malignancy. Fortunately, early symptoms such as postmenopausal bleeding often lead to timely detection, and localized disease treated surgically achieves a 5-year survival rate of up to 95% (5). However, disease recurrence carries a poor prognosis and represents a leading cause of mortality in EC patients (6). Therefore, accurate prediction of recurrence and individualized risk stratification are critical not only for tailoring treatment strategies and follow-up management to reduce recurrence, but also for avoiding overtreatment, alleviating financial burden, and optimizing healthcare resource allocation (7). This clinical landscape underscores the urgent need to explore novel prognostic biomarkers and develop more refined risk assessment systems.

Currently, the prediction of EC recurrence primarily relies on clinicopathological parameters such as age, FIGO stage, histologic type and grade, depth of myometrial invasion, and lymphovascular space invasion, as well as molecular biomarkers (8). According to the 5th edition World Health Organization (WHO) classification, EC can be categorized into four molecular subtypes: POLE-mutant (12%), mismatch repair-deficient (30%), p53-abnormal (18%), and no specific molecular profile (NSMP, 40%). The latter two subtypes collectively account for 70% of cases (9, 10). However, the current molecular subtyping system still has limitations, including high testing costs and limited diagnostic efficacy, underscoring the need to develop novel auxiliary predictive biomarkers.

Recent studies have revealed that insulin-like growth factor 2 mRNA-binding protein 2 (IGF2BP2) plays a significant role in various malignancies, such as ovarian, esophageal, pancreatic, and hepatocellular carcinomas. Its overexpression is closely associated with tumor progression and poor prognosis (11–14). Although IGF2BP2 has been recognized as a potential prognostic factor in multiple cancers, its role in the prognostic stratification of EC remains insufficiently investigated and warrants further exploration.

Considering this, the present study addresses the need for precision medicine in EC by focusing on the correlation between IGF2BP2 expression levels and patient recurrence risk. By integrating IGF2BP2 expression with key clinicopathological parameters, we developed a nomogram model to quantitatively assess recurrence risk. This model aims to provide a more accurate and practical tool for individualized prognosis evaluation in patients with EC.

## Materials and methods

### Study population

This multicenter retrospective cohort study enrolled patients with stage I–III EC who underwent primary surgical treatment between January 2016 and January 2023 at the First Affiliated Hospital of Chongqing Medical University (training cohort, n = 545) and Liangjiang Hospital of Chongqing Medical University and Women and Children’s Hospital of Chongqing Medical University (validation cohort, n = 315). The inclusion criteria were as follows (1): receipt of standardized surgical treatment, consisting of total hysterectomy with bilateral salpingo-oophorectomy, with the performance of systematic lymph node assessment determined by pathological risk features (15); The extent of lymphadenectomy (pelvic only or combined with para-aortic) was determined by comprehensive preoperative and intraoperative evaluation. Established criteria for identifying patients at low risk of lymph node metastasis include: (a) myometrial invasion less than 50%; (b) tumor diameter less than 2 cm; and (c) histologic grade G1 or G2. However, accurately determining these parameters before final pathology can often be challenging. When possible, intraoperative frozen section evaluation by gynecologic pathologists for assessing myometrial invasion and cervical involvement may guide decision-making—for instance, omitting systematic lymphadenectomy in cases confirmed to have no myometrial invasion or cervical involvement (16, 17) (2); availability of complete preoperative baseline data; and (3) completion of standard preoperative laboratory tests (including complete blood count, liver and renal function, coagulation profile, and tumor markers such as CA125 and HE4) and imaging studies (chest and abdominal CT, and pelvic MRI). Exclusion criteria included (1): non-standard surgical treatment (2); receipt of neoadjuvant therapy prior to surgery (3); incomplete clinical data (4); loss to follow-up (5); history of other malignant tumors; and (6) significant inflammatory or immune system diseases.

### Treatment

Adjuvant treatment regimens were individualized based on patients’ postoperative pathological characteristics. Radiotherapy was administered to those exhibiting at least one high-risk feature (18): age ≥60 years, specific histologic types (e.g., serous or clear cell carcinoma), high-grade tumor (G3), deep myometrial invasion (≥1/2), cervical stromal involvement, or lymphovascular space invasion. The radiotherapy regimen consisted of either vaginal brachytherapy (total dose 22–24 Gy in 4 fractions) or pelvic external beam radiotherapy (total dose 45–50 Gy in conventional fractions). For patients with FIGO stage III or higher disease, specific pathologic types, or G3 tumors with deep myometrial invasion, the carboplatin-paclitaxel regimen was recommended as the first-line systemic chemotherapy, administered over six 21-day cycles (15, 19).

### Follow-up

All patients were managed under a standardized postoperative surveillance protocol: quarterly in the first two years, semiannually during years 3–5, and annually after five years. Follow-up assessments included (1): baseline evaluations, which consisted of detailed history-taking and pelvic-rectal examination (2); biomarker tests, such as CA125, when clinically indicated; and (3) diagnostic imaging (ultrasound, CT, or MRI) for suspected recurrence or metastasis. The study follow-up concluded in January 2025. Given that approximately 70%–80% of endometrial cancer recurrences occur within the first three years postoperatively, all surviving patients had completed at least 36 months of surveillance, excluding those lost to follow-up or deceased (18).

### Recurrence

Recurrence is strictly defined as meeting both of the following criteria (1): independent confirmation by at least two gynecologic oncologists; and (2) objective evidence from at least one of the following: ① confirmed lesions on cross-sectional imaging (CT/MRI/PET-CT), ② histopathologic confirmation of malignant cells, or ③ persistently elevated serum tumor markers (e.g., CA125) after excluding other etiologies (6). Based on anatomic site, recurrences were categorized as either locoregional (including vaginal vault recurrence and pelvic sites such as the vesicorectal space) or distant metastasis (encompassing para-aortic lymph node involvement, peritoneal dissemination, or hematogenous spread to solid organs like liver, lung, or bone) (20). Recurrence-free survival (RFS) was calculated from the date of comprehensive staging surgery to the first occurrence of either pathologically or radiologically confirmed recurrence. Patients without recurrence were censored at their last follow-up date. Overall survival (OS) was measured from the initial surgery date until death from any cause. Surviving patients were censored at the study’s cutoff date, whereas those lost to follow-up were censored at the last documented contact (21).

### Data collection

We retrospectively collected clinicopathological data, including: 1) baseline characteristics: age and body mass index; 2) tumor features: FIGO stage, maximum tumor diameter, histologic subtype and grade, cervical stromal invasion, myometrial invasion depth (<50% or ≥50%), and lymphovascular space invasion. Formalin-fixed paraffin-embedded tissue specimens were obtained from the pathology department for subsequent immunohistochemical analysis of IGF2BP2 protein expression.

### Data pre-processing and quality control

**Figure 1:**
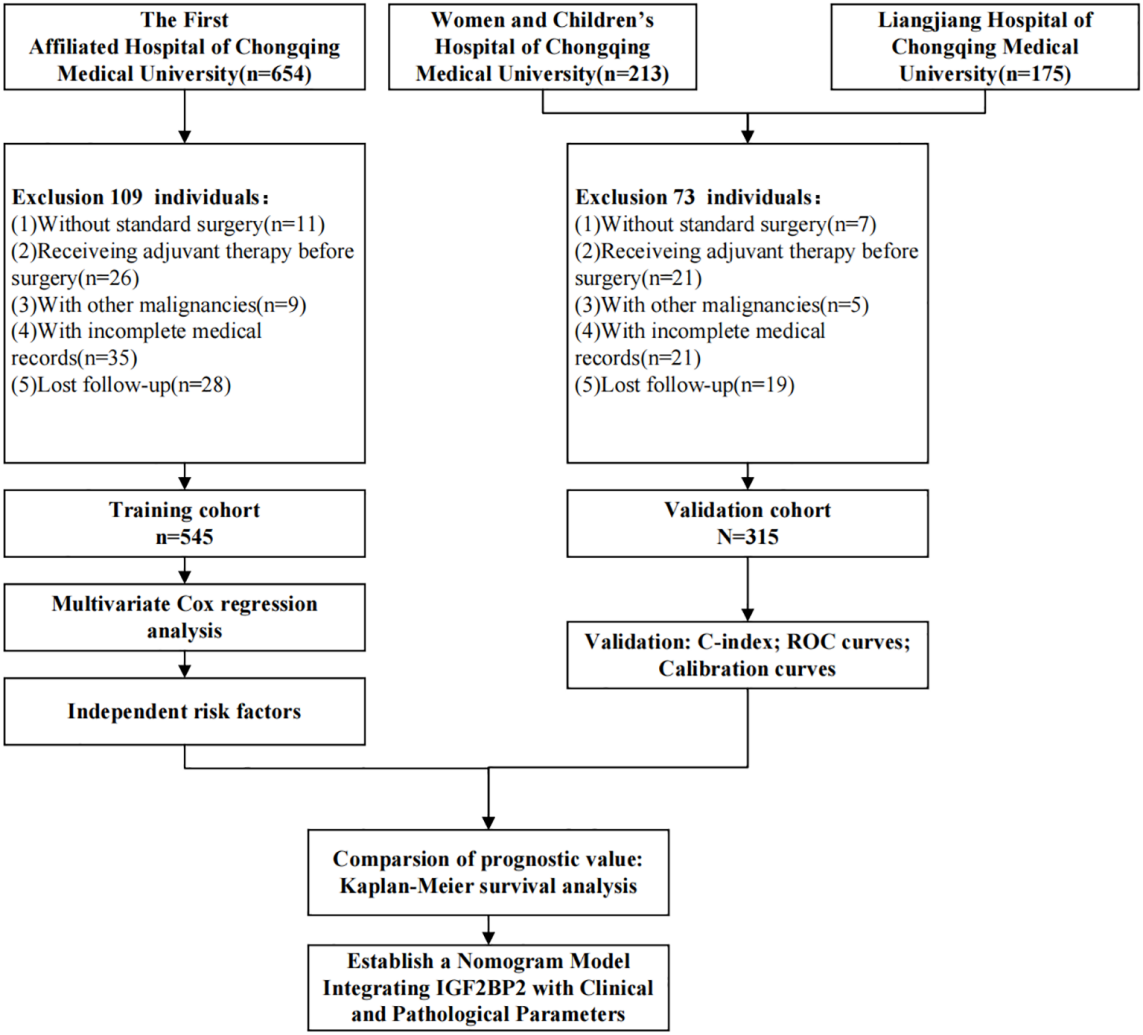
Flow chart of patient selection for EC study.

Our pre-processing and quality control pipeline comprised the following systematic steps to ensure data integrity and reproducibility: (1) Harmonization and standardization of multi-center data: To standardize the data collected from multiple sites, a centralized harmonization procedure was implemented. All categorical variables—such as FIGO stage and histological type—were recoded using standardized international classification systems (the FIGO 2009 staging system and the WHO 2020 classification for histology). Continuous variables, including age and BMI, were retained in their original numerical units without categorization at the pre-processing stage. (2) Exclusion of cases with missing critical variables: As detailed in the study flowchart (**Figure 1**), patients with missing data for any of the key variables ultimately included in the final nomogram model were excluded prior to model development. (3) Quality control and inter-rater reliability assessment for immunohistochemical scoring: Two expert gynecological pathologists independently assessed all IGF2BP2 and p53 slides while blinded to clinical outcomes. Inter-rater consistency was formally evaluated using the intraclass correlation coefficient (ICC). Discrepancies exceeding 10% were resolved through a consensus review using a multi-headed microscope. (4) Appropriate transformation of variables for clinical application: To align with established clinical practice and enhance the utility of the nomogram, specific continuous variables were dichotomized using validated or commonly accepted thresholds. Age was categorized as ≥60 vs. <60 years. Serum CA125 was dichotomized at 35 U/mL. The semi-quantitative IGF2BP2 immunohistochemical score was converted to a binary variable (high vs. low expression) using the median score of the training cohort as the optimal threshold, as determined by ROC analysis. (5) Verification of cohort balance: Baseline characteristics between the training and validation cohorts were compared using Chi-square tests (categorical) and t-tests (continuous) to ensure comparability.

### Tissue processing and pathological analysis

Postoperative tissue specimens were fixed in formalin and uniformly processed for paraffin embedding, sectioning, and H&E staining by the pathology departments across all participating centers to identify carcinoma regions.

Pathological evaluation encompassed tumor location, maximum diameter, histologic type and grade, depth of myometrial invasion, lymphovascular space invasion (LVSI), and cervical stromal involvement. Histologic classification followed established criteria (21, 22), categorizing tumors as G1/G2 endometrioid adenocarcinoma, G3 endometrioid adenocarcinoma, or non-endometrioid subtypes (including serous and clear cell carcinomas) (21, 22).

### Immunohistochemistry

**Figure 2:**
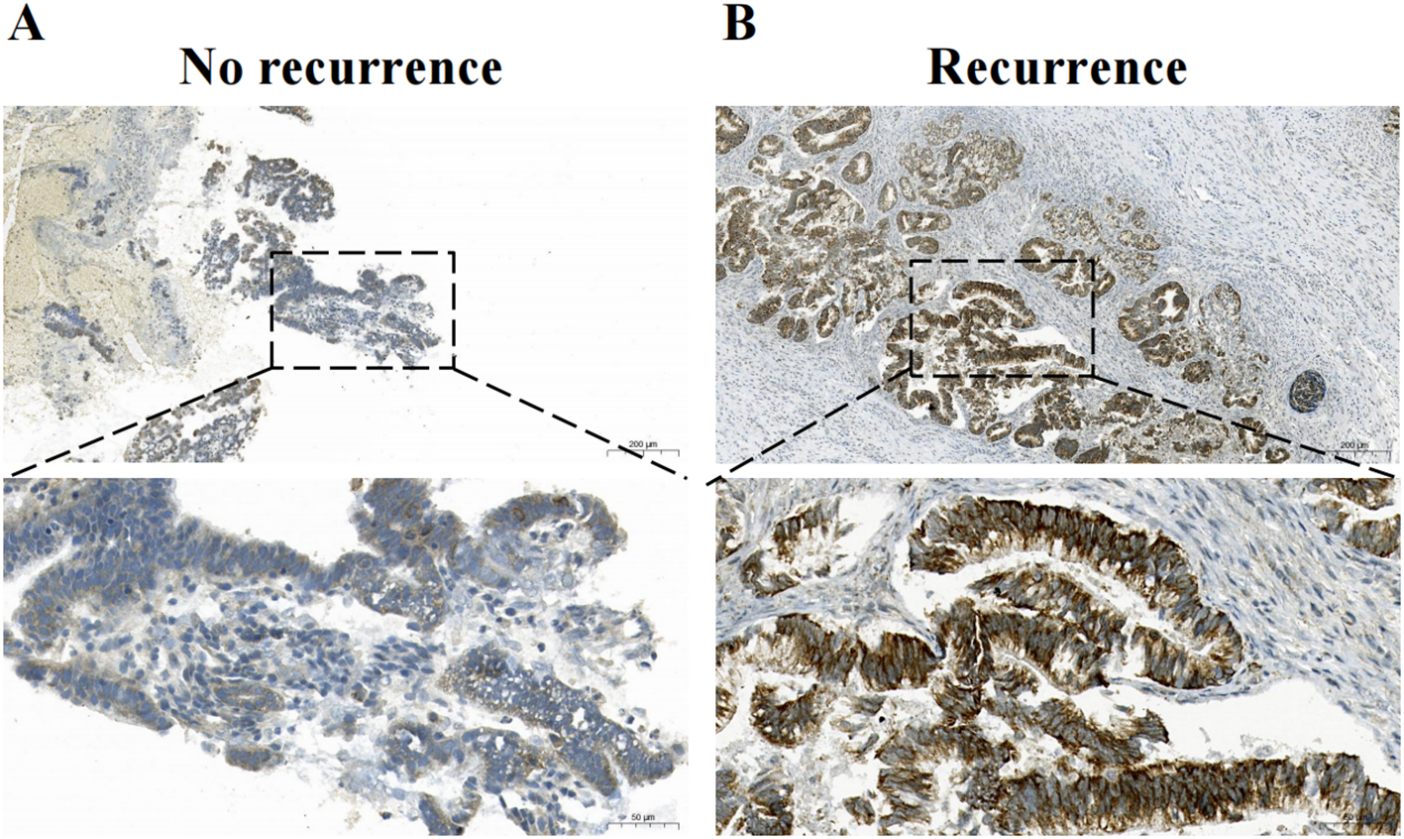
Representative photomicrographs of IGF2BP2 immunohistochemical staining. **(A)** Low IGF2BP2 expression in a case without recurrence. **(B)** High IGF2BP2 expression in a case with tumor recurrence.

Tissue sections were processed following standard Immunohistochemistry (IHC) protocols including antigen retrieval in citrate buffer (pH 6.0) and peroxidase blocking. Primary antibody incubations were performed with anti-IGF2BP2 (1:300, 4 °C overnight) and anti-p53 (clone DO7, 1:200) using an automated stainer. Detection was completed with HRP-conjugated secondary antibody and DAB visualization (21).Two pathologists independently assessed protein expression. P53 nuclear expression was quantified as percentage of positive cells (0-100%). Based on established molecular classification criteria for endometrial carcinoma, cases were categorized into three patterns with defined biological and clinical implications: p53-abnormal (overexpression pattern)-≥75% of tumor nuclei exhibiting strong, diffuse staining; p53-abnormal (null pattern)-complete absence (0%) of nuclear staining with appropriate internal positive controls; or p53-wild-type—1-75% of tumor nuclei showing heterogeneous staining intensity. This classification correlates with TP53 mutation status and informs molecular subtyping. IGF2BP2 cytoplasmic staining was semi-quantitatively scored based on intensity and distribution. Immunohistochemical assessment of IGF2BP2 employed a semi-quantitative scoring system (range 1–4), derived by multiplying the percentage of positively stained tumor cells by the staining intensity (graded on a 1–3 scale). This yielded final scores categorized as follows: 1 = negative, 2 = weak positivity, 3 = moderate positivity, and 4 = strong positivity. Using the median score of the training cohort as the optimal threshold, specimens with scores ≥2.5 were classified into the high-expression group for subsequent nomogram incorporation. IGF2BP2 expression was markedly higher in recurrent tumors compared to recurrence-free cases, as evidenced by representative immunohistochemical staining (**Figure 2**). Inter-observer differences in H-scores ≤10% were resolved by averaging the two independent assessments. For cases with discrepancies >10% (n=37, 4.3%), a formal consensus review was conducted: both pathologists jointly re-examined the slides using a multi-headed microscope and, through unblinded discussion guided by the standardized criteria, reached a definitive classification. This two-tier approach ensured consistent application of the scoring system across all specimens (23).

### Study design and statistical analysis

This retrospective cohort study included 545 endometrial cancer patients from the First Affiliated Hospital of Chongqing Medical University (January 2016-January 2023) as the training cohort, and 315 patients from the Women and Children’s Hospital and Liangjiang Hospital of Chongqing Medical University as the validation cohort. Categorical variables were presented as frequencies (percentages) and compared using χ² or Fisher’s exact tests; continuous variables were expressed as mean ± standard deviation and analyzed with independent samples t-tests. Statistical significance was declared for findings with p-values below 0.05.

Univariable Cox regression was first employed to identify clinicopathological parameters and IGF2BP2 expression levels associated with RFS (P < 0.05). Significant variables were subsequently incorporated into a multivariable Cox model to determine independent prognostic factors. A nomogram was constructed to predict 1-, 3-, and 5-year RFS probabilities. The optimal cutoff value for IGF2BP2 expression was determined by receiver operating characteristic (ROC) curve analysis, with the maximum Youden index (sensitivity + specificity - 1) as the criterion (24). Patients were stratified into high- and low-risk groups based on the predicted 3-year recurrence-free survival probability derived from the model. Kaplan-Meier survival curves were generated, and between-group differences were assessed using the log-rank test.

Calibration curves were plotted in both the training and validation cohorts to evaluate the agreement between predicted and observed outcomes. The concordance index (C-index) was calculated to assess model discrimination, with values of 0.5–0.6 indicating limited, 0.6–0.7 moderate, and >0.8 strong discriminatory ability. All statistical analyses were performed using SPSS (version 25.0, IBM Statistics, Chicago, IL, USA) and R software (version 4.0.3, http://www.r-project.org), with a two-sided P < 0.05 considered statistically significant (25).

## Results

### Patient characteristics

This study enrolled a total of 860 EC patients, with 545 cases from The First Affiliated Hospital of Chongqing Medical University comprising the training cohort and 315 cases from the Liangjiang Hospital of Chongqing Medical University and Women and Children’s Hospital of Chongqing Medical University forming the validation cohort (**Figure 1**). As summarized in **Table 1**, the two cohorts demonstrated comparable distributions across all documented clinicopathological features-including age, body mass index(BMI), FIGO stage, histologic type, grade, cervical invasion, myometrial invasion depth, LVSI, adjuvant treatment, CA125 and IGF2BP2 expression level (all P > 0.05)-indicating well-balanced baseline characteristics between the groups.

**Table 1:** Baseline clinicopathological characteristics of EC patients in the training and validation cohorts.

Variable	Training cohort, N = 545	%	Validation cohort, N = 315	%	p value
Age (yrs) Mean (± SD)	54.01 (± 9.44)		54.01(± 9.52)	–	0.995
BMI (kg/m2) Mean (± SD)	24.55 ± 3.81		24.34 ± 3.74	–	0.396
FIGO stage I II III	370 59 116	67.9 10.8 21.3	212 39 64	67.3 12.4 20.3	0.783
Histologic type G1-G2 Endometrioid G3 Endometrioid Non-endometrioid	396 61 88	72.7 11.2 16.1	232 38 46	73.3 12.1 14.6	0.799
Cervical stromal invasion No Yes	465 80	85.3 14.7	261 54	82.9 17.1	0.337
Myometrial invasion <1/2 ≥1/2	398 147	73.0 27.0	232 82	74.0 26.0	0.784
LVSI negative positive	412 133	75.6 24.4	242 73	76.8 23.2	0.684
Serum CA125 (U/ml) <35 ≥35	403 142	73.9 26.1	232 83	73.7 26.3	0.925
Adjuvant treatment No Yes	195 350	35.8 64.2	103 212	32.7 67.3	0.360
IGF2BP2 expression low- expression high- expression	372 173	68.3 31.7	212 103	67.3 32.7	0.772

BMI, body mass index; FIGO, International Federation of Gynecology and Obstetrics; LVSI, lymphovascular space invasion; IGF2BP2, insulin-like growth factor 2 mRNA-binding protein 2.

### Independent risk factors for recurrence in endometrial cancer

As indicated in **Table 2**, the multivariable Cox regression analysis revealed FIGO stage (p = 0.001), depth of myometrial invasion (p = 0.004), histologic type (p = 0.001), LVSI (p = 0.007), CA125 level (p = 0.001), p53 expression status (p = 0.013), and IGF2BP2 expression (p < 0.001) as independent prognostic factors for RFS in EC patients.

**Table 2:** Univariable and multivariable cox regression analyses of RFS in the training cohort.

Variables	Univariate analysis			Multivariate analysis		
	Hazard ratio	95% CI	P value	Hazard ratio	95% CI	P value
Age (yrs) (≥60vs <60)	3.452	2.205-5.403	<0.001	1.841	1.117-3.034	0.017
FIGO stage I II III	- 3.792 9.973	- 1.804-7.968 5.849-17.005	<0.001 <0.001 <0.001	1.000 2.250 2.975	- 0.782-6.472 1.560-5.673	0.004 0.133 0.001
Histologic type G1-G2 Endometrioid G3 Endometrioid Non-endometrioid	- 3.279 6.188	- 1.738-6.184 3.771-10.152	<0.001 <0.001 <0.001	- 1.845 2.616	- 0.933-3.649 1.497-4.573	0.003 0.078 0.001
Cervical stromal invasion (Yes vs No)	2.305	1.396-3.807	0.001	1.136	0.551-2.340	0.730
Myometrial invasion (≥1/2 vs <1/2)	5.263	3.318-8.350	<0.001	2.134	1.274-3.575	0.004
LVSI (positive vs negative)	4.523	2.883-7.096	<0.001	2.014	1.212-3.349	0.007
CA125 (≥35 vs <35)	2.877	1.840-4.500	<0.001	1.658	1.015-2.708	0.001
P53 (positive vs negative)	1.013	1.006-1.020	<0.001	1.824	1.135-2.933	0.013
Adjuvant treatment (Yes vs No)	2.232	1.287-3.870	0.004	0.541	0.289-1.014	0.055
IGF2BP2 (high- expression vs low- expression)	3.737	2.363-5.909	<0.001	2.730	1.687-4.417	0.000

FIGO, International Federation of Gynecology and Obstetrics; LVSI, lymphovascular space invasion; IGF2BP2, insulin-like growth factor 2 mRNA-binding protein 2; CI, confidence interval; HR, hazard ratio; RFS, recurrence-free survival.

**Figure 3:**
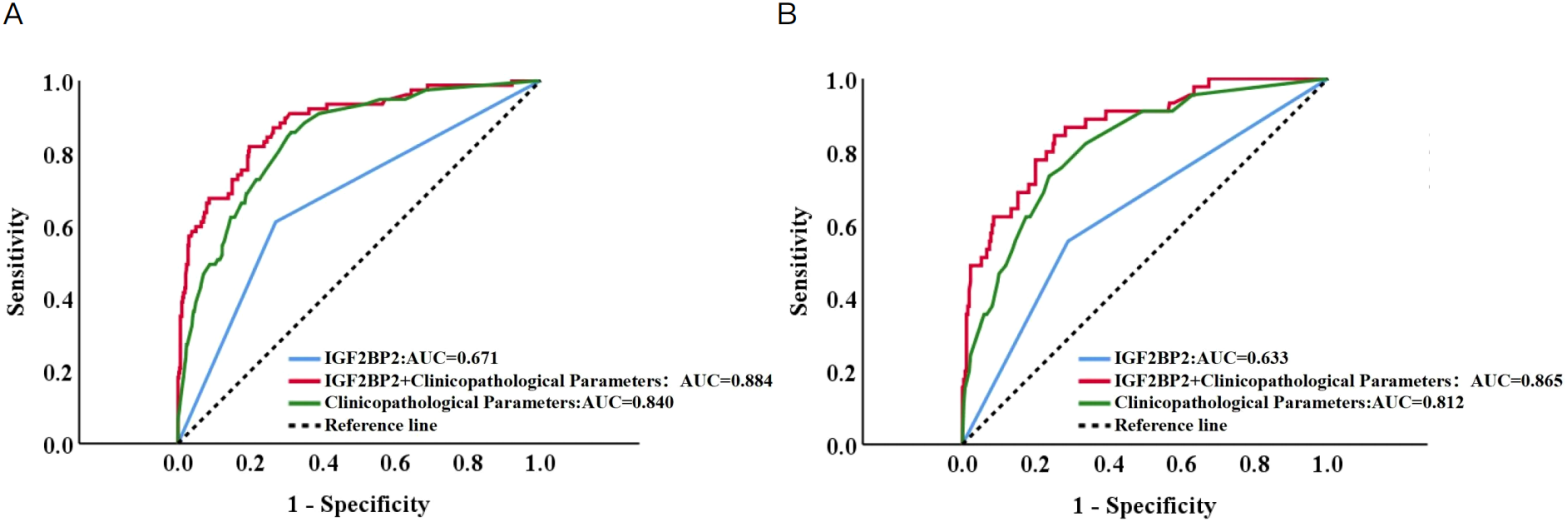
Receiver operating characteristic (ROC) curves of IGF2BP2, individual clinicopathological parameters, and their combination in the training and validation cohorts. (**A**) Training cohort: ROC curves of IGF2BP2 alone, individual clinicopathological parameters, and their combination. **(B)** Validation cohort: ROC curves of IGF2BP2 alone, individual clinicopathological parameters, and their combination.

In the training cohort, the nomogram demonstrated superior predictive performance, achieving an area under the ROC curve (AUC) of 0.884 (95% CI: 0.841–0.927), which outperformed both the clinicopathological parameter-based model (AUC = 0.840, 95% CI: 0.794–0.886) and IGF2BP2 alone (AUC = 0.671, 95% CI: 0.603–0.738). This discriminative ability was consistently maintained in the validation cohort, with AUCs of 0.865 (95% CI: 0.808–0.922) for the nomogram, 0.812 (95% CI: 0.776–0.889) for the clinical model, and 0.633 (95% CI: 0.543–0.724) for IGF2BP2 alone (**Figure 3**). It yielded a C-index of 0.884 (95% CI: 0.841–0.927) in the training cohort, which further increased to 0.865 (95% CI: 0.808–0.922) in the validation cohort, indicating robust performance.

**Figure 4:**
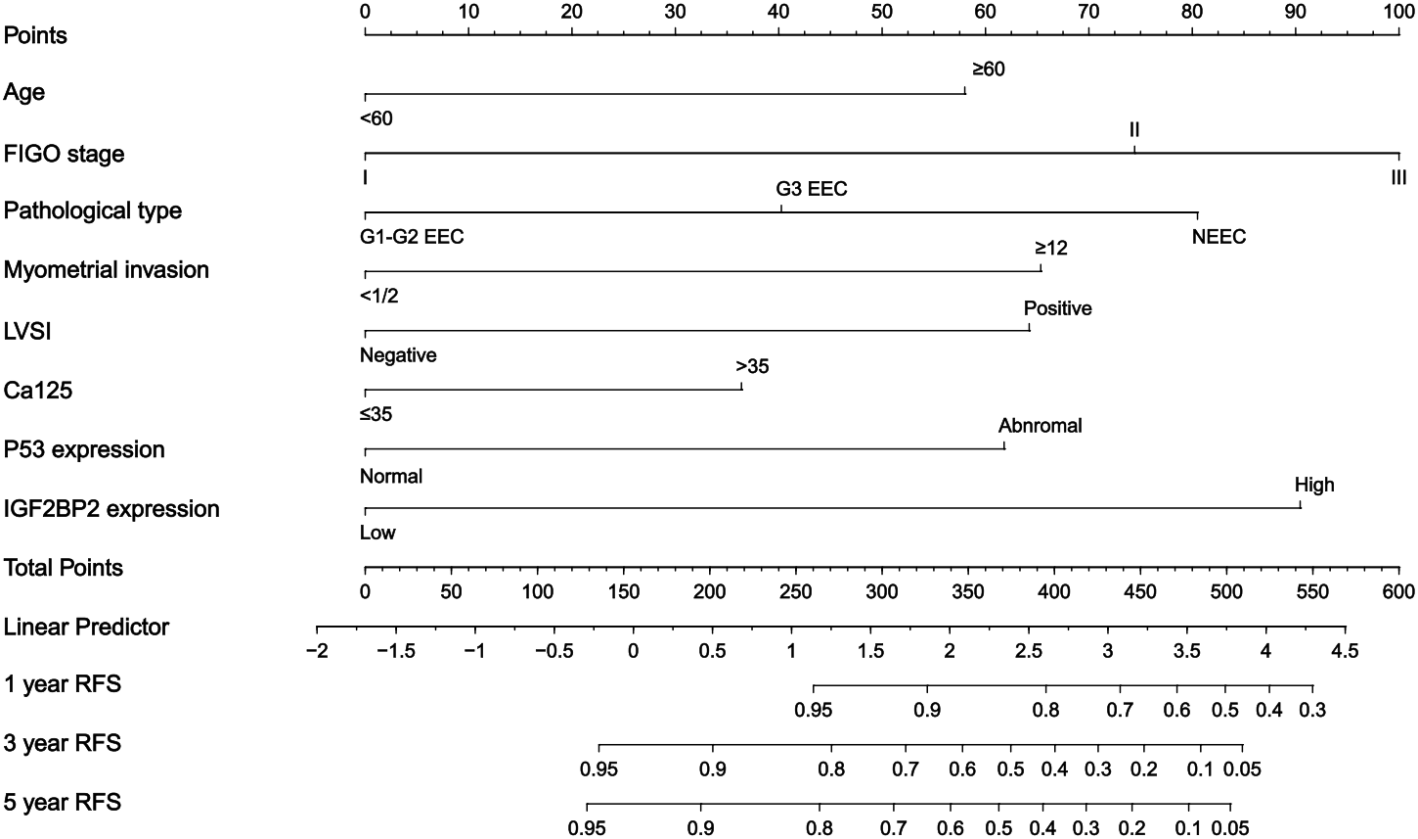
Nomogram for predicting 1-, 3-, and 5-year RFS in EC patients. To estimate the probability of recurrence, locate the patients’ grade on the “grade” axis. Draw a straight line up to the “point” axis to determine the points for grade. Repeat the process for each of the remaining axes, drawing a straight line each time to the “point” axis. Add the points received from each variable and locate this number on the “total point” axis. A straight line is drawn down from the “total point” axis to the “probability of 1-, 3-, and 5-year RFS” axis to determine the risk of recurrence in patients.

Based on multivariable Cox regression analysis, we developed a nomogram (**Figure 4**) integrating clinicopathological parameters-including FIGO stage, histologic type and grade, cervical stromal invasion, myometrial invasion, LVSI, and adjuvant therapy-along with IGF2BP2 expression to predict recurrence-free survival (RFS) in endometrial cancer patients. By summing the points assigned to each predictor in the nomogram, individualized recurrence risk can be quantitatively assessed.

**Figure 5:**
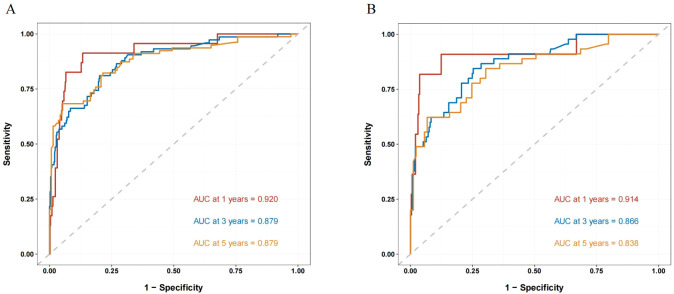
Time-dependent ROC curves for predicting 1-, 3-, and 5-year RFS. **(A)** Internal validation in the training cohort. **(B)** External validation in the independent validation cohort. The curves demonstrate the model’s consistent ability to distinguish between patients who will experience recurrence and those who will not at 1, 3, and 5 years. The high AUC values across both cohorts confirm the generalizability and temporal stability of the prognostic model.

**Figure 6:**
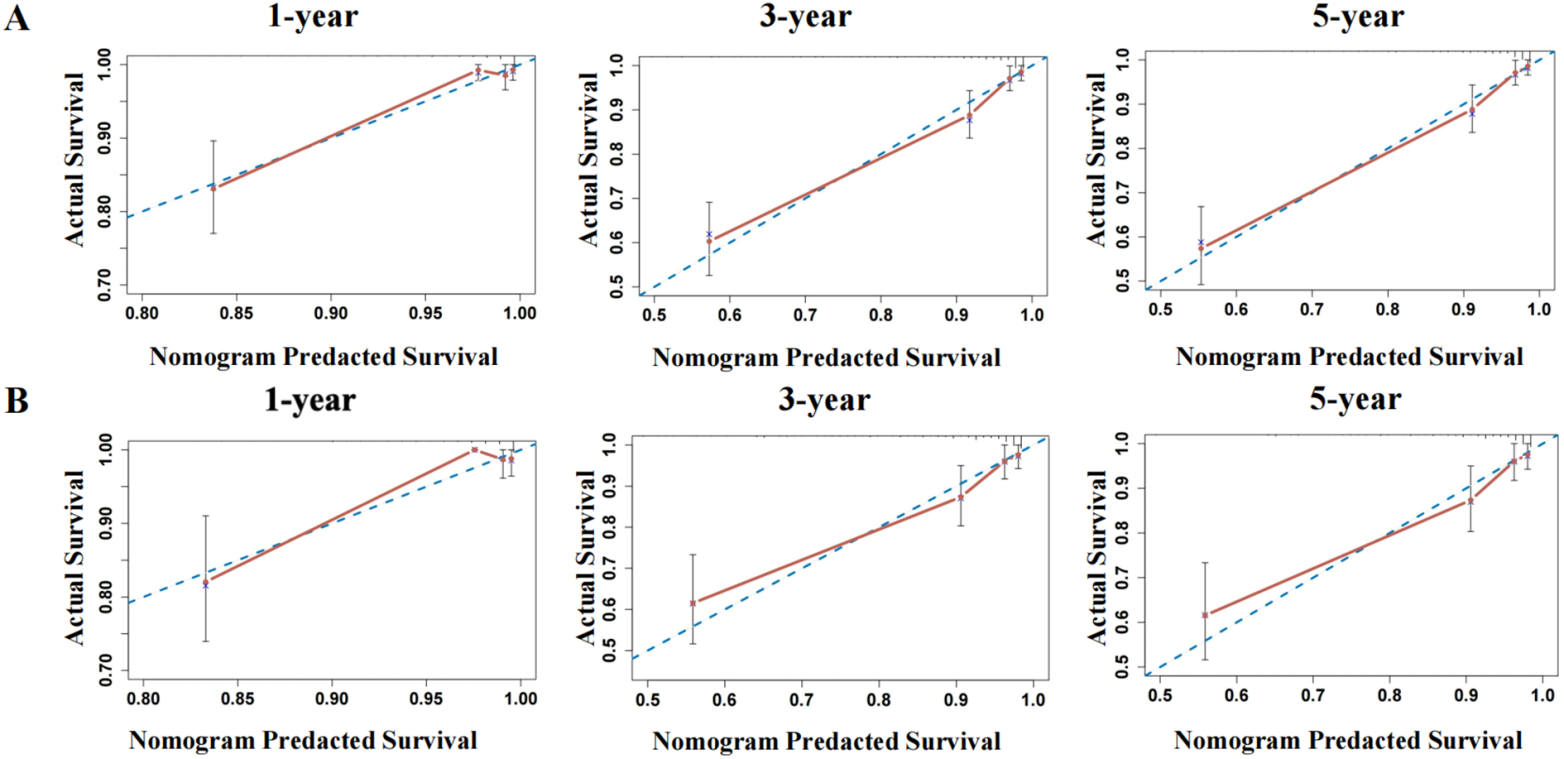
Calibration curves for 1-, 3-, and 5-year RFS in the training and validation cohorts. **(A)** The calibration curve for internal validation of the nomogram model for predicting the 1-, 3-, and 5-year RFS in EC; **(B)**The calibration curve for external validation of the nomogram model for predicting the 1-, 3-, and 5-year RFS in EC (The blue dotted line: reference line; The red solid line: the prediction curve given by the model).

The nomogram exhibited exceptional discriminative capacity for forecasting 1-, 3-, and 5-year RFS, achieving AUCs of 0.920, 0.879, and 0.879 in the training cohort and 0.914, 0.866, and 0.838 in the validation cohort, respectively. These outcomes affirm its robust performance across various postoperative intervals, particularly sustaining high predictive accuracy during the 3-year peak recurrence period, thereby offering a quantitative foundation for customizing individualized follow-up strategies. The nomogram-predicted RFS rates exhibited excellent concordance with actual observations in both the training and validation cohorts (**Figure 5**), suggesting favorable calibration and clinical predictive accuracy of the model. The calibration curves demonstrated exceptional agreement between the model-predicted probabilities and the observed outcomes in both the training and validation cohorts (**Figure 6**), indicating that the model is well-calibrated.

### Determination of the optimal risk threshold and its prognostic value

**Figure 7:**
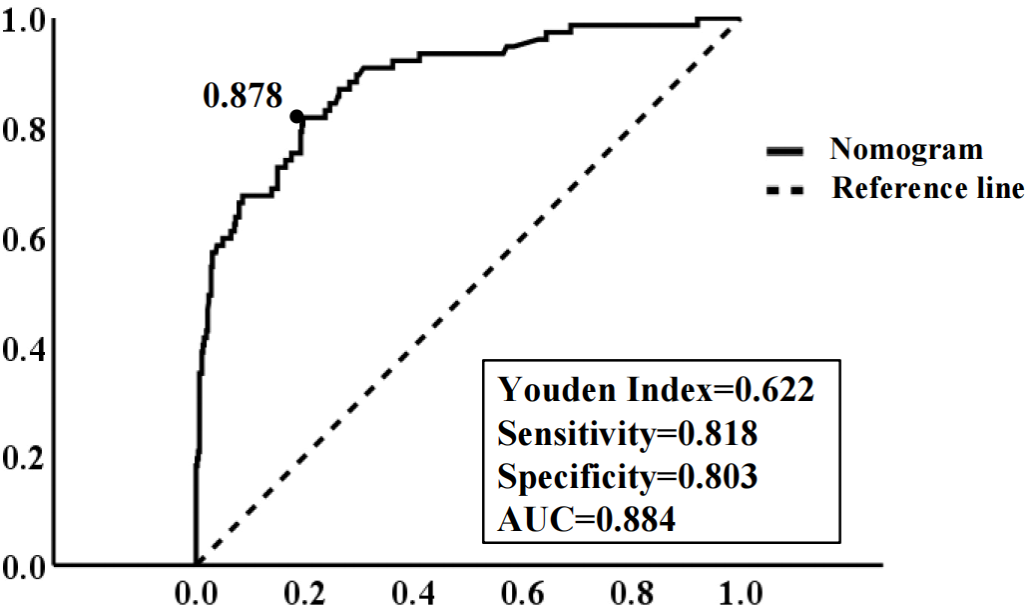
ROC curve of the model for predicting EC recurrence. The area under the curve at the “black dot” is the largest, which suggests that the optimal threshold value of the probability of predicted by the model is 0.878 (area under the curve = 0.884; sensitivity, 81.8%; specificity, 80.3%); (Notes: dotted line: reference line; solid line: the ROC curve of the model.).

ROC analysis identified an optimal IGF2BP2 cutoff value of 0.878 for predicting endometrial cancer recurrence, with an area under the curve (AUC) of 0.884, sensitivity of 81.8%, and specificity of 80.3% (**Figure 7**). This threshold was determined by maximizing the Youden index (sensitivity + specificity - 1), reflecting the model’s strong discriminative capacity in identifying high-risk patients.

Based on ROC analysis and the maximum Youden index, the optimal risk threshold for predicting EC recurrence in the training cohort was determined to be 0.878 (**Figure 7**). Patients were stratified into low-risk (3-year RFS ≥ 0.878) and high-risk (3-year RFS < 0.878) groups. Kaplan-Meier analysis revealed significantly lower 3- and 5-year recurrence-free survival rates in high-risk patients (79.80% [95% CI: 73.72–85.88%] and 74.00% [95% CI: 67.53–80.47%], respectively) compared to low-risk patients (both 91.90% [95% CI: 89.16–94.64%]) (P < 0.001). Similarly, overall survival was significantly reduced in the high-risk group (3-year: 80.90% [95% CI: 75.02–86.78%]; 5-year: 79.70% [95% CI: 79.64–85.78%]) versus the low-risk group (3-year: 95.40% [95% CI: 93.24–97.56%]; 5-year: 95.10% [95% CI: 92.94–97.26%]) (**Tables 3**, **4**). This survival disparity was consistently validated in the independent cohort.

**Table 3:** Comparison of RFS and OS between low and high IGF2BP2 expression groups in the training cohort.

Group	Number of patients (n=545)	Recurrence (n=77)	3-year RFS (95% CI)	5-year RFS (95% CI)	p value	Death (n=61)	3-year OS (95%CI)	5-year OS (95%CI)	p value
low-IGF2BP2 expression	372	30	91.90% (89.16-94.64%)	91.90% (89.16-94.64%)	<0.001	22	95.40% (93.24-97.56%)	95.10% (92.94-97.26)	<0.001
High-IGF2BP2 expression	173	47	79.80% (73.72-85.88%)	74.00% (67.53-80.47%)		39	80.90% (75.02-86.78%)	79.70% (79.64-85.78)	

CI, confidence interval; IGF2BP2, insulin-like growth factor 2 mRNA-binding protein 2; RFS, recurrence-free survival; OS, overall survival.

**Table 4:** Comparison of RFS and OS between low and high IGF2BP2 expression groups in the validation cohort.

Group	Number of patients (n=315)	Recurrence (n=45)	3-year RFS (95% CI)	5-year RFS (95% CI)	p value	Death (n=61)	3-year OS (95%CI)	5-year OS (95%CI)	p value
low-IGF2BP2 expression	212	20	90.60% (86.68-94.52%)	90.60% (86.68-94.52%)	<0.001	13	94.80% (91.86-97.74%)	93.70% (90.37-97.03%)	<0.001
High-IGF2BP2 expression	103	25	75.70% (67.47-83.93%)	75.70% (67.47-83.93%)		21	82.50% (75.25-89.75%)	79.20% (71.16-87.24%)	

CI, confidence interval; IGF2BP2, insulin-like growth factor 2 mRNA-binding protein 2; RFS, recurrence-free survival; OS, overall survival.

**Figure 8:**
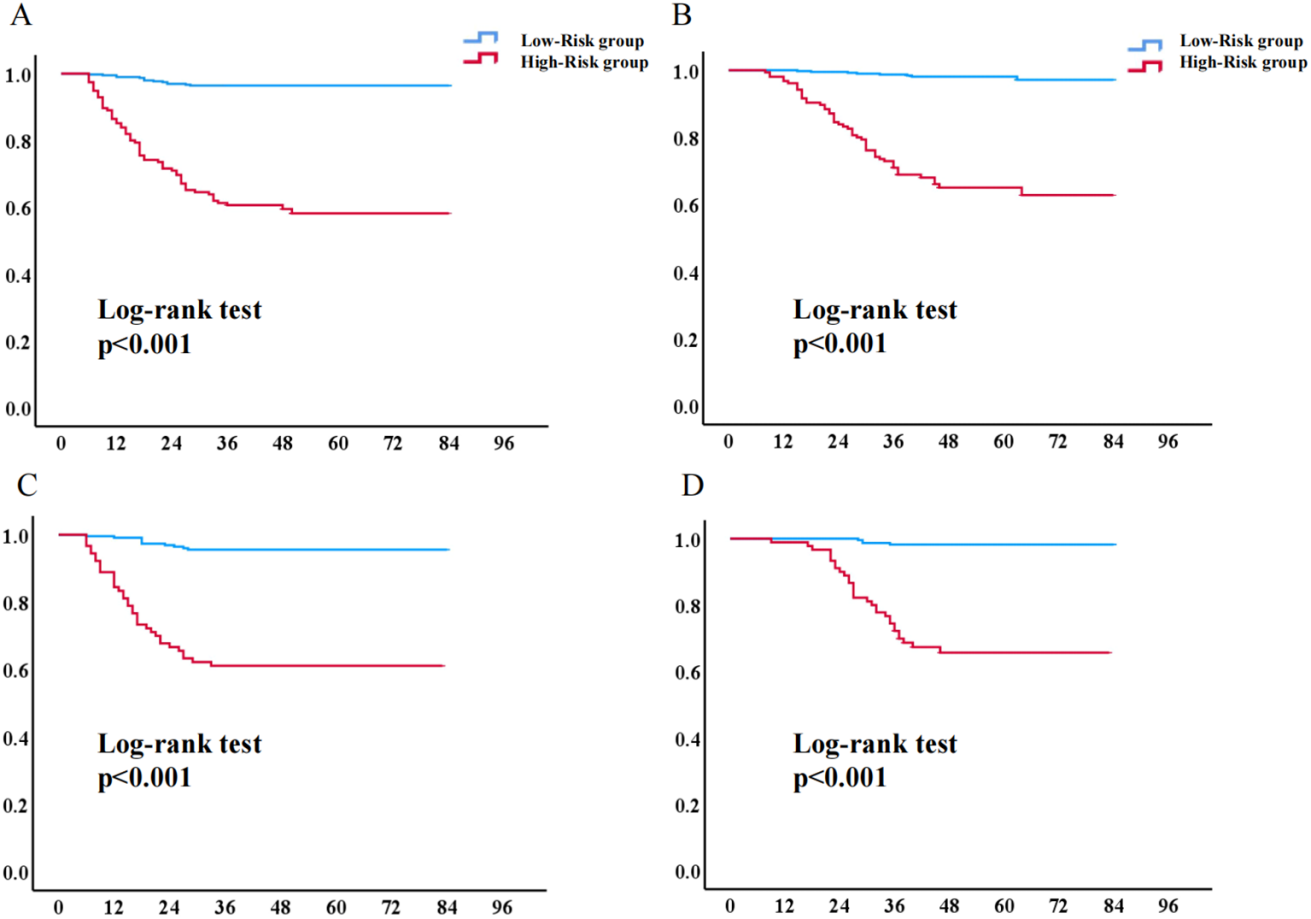
Kaplan-Meier survival curves for high- and low-risk patient groups. **(A)** Recurrence-free survival curve of the low-risk and high-risk groups in the training cohort; **(B)** Overall survival curve of the low-risk and high-risk groups in the training cohort. **(C)** Recurrence-free survival curve of the low-risk and high-risk groups in the validation cohort. **(D)** Overall survival curve of the low-risk and high-risk groups in the validation cohort. (Notes: the red line: high-risk group; the blue line: low-risk group.).

Utilizing the optimal cutoff value of 0.878 obtained from the model, patients in both the training and validation cohorts were categorized into high-risk (predicted risk probability ≥ 0.878) and low-risk (predicted risk probability < 0.878) groups. Kaplan-Meier analysis revealed significantly shorter RFS and OS in the high-risk group compared to the low-risk group (**Figure 8**).

To investigate the association between IGF2BP2 expression and key pathological markers in endometrial carcinoma, we compared p53, ER, and PR status between IGF2BP2 low- and high-expression groups. As shown in **Supplementary Table S1**, tumors with high IGF2BP2 expression exhibited a significantly higher frequency of abnormal p53 compared to the low-expression group 45.7% vs 30.4%, p=0.001). Furthermore, high IGF2BP2 expression was strongly associated with ER-negative (27.7% vs 14.2%, p<0.001) and PR-negative (33.5% vs 17.2%, p<0.001) status. These findings suggest that elevated IGF2BP2 expression may be linked to a hormone receptor-negative and p53-aberrant molecular phenotype in endometrial carcinoma.

## Discussion

EC is a common gynecologic malignancy with a continuously rising global incidence (3, 26). Over the past three decades, its overall incidence has increased by 132%, with approximately 417,000 new cases reported worldwide in 2020 (27). Given the suboptimal outcomes for patients with advanced or recurrent disease, investigating its molecular mechanisms and identifying precise predictive biomarkers are of great importance.

IGF2BP2, an RNA-binding protein, has demonstrated prognostic regulatory potential in multiple malignancies (28). This study confirms IGF2BP2 as an independent risk factor for EC recurrence-patients with high IGF2BP2 expression exhibited significantly shorter overall survival and RFS, consistent with recent findings (29). Based on this, we developed a nomogram integrating IGF2BP2 with clinicopathological parameters. This model visually represents the weight of each predictor, enabling quantitative assessment of individualized recurrence risk. For example, a patient with multiple high-risk features-age ≥60 years (56 points), FIGO stage II (74 points), G3 endometrioid carcinoma (40 points), myometrial invasion ≥1/2 (64 points), lymphovascular space invasion (63 points), CA125 >35 U/mL (36 points), p53 positivity (62 points), and high IGF2BP2 expression (90 points)-would have a total score of 485 points, corresponding to 1-, 3-, and 5-year RFS probabilities of 55%, 8%, and 6%, respectively. The high- and low-risk groups, defined by the model’s optimal threshold, showed significant prognostic differences in both training and validation cohorts. Moreover, the model demonstrated favorable predictive accuracy and consistency in calibration curves and time-dependent ROC analyses.

The core innovation of this study lies in the effective integration of the novel molecular marker IGF2BP2 with conventional clinical indicators. The resulting model achieved AUCs of 0.884 and 0.865 in the training and validation cohorts, respectively, significantly outperforming prediction systems based solely on clinical parameters. This tool can serve as a valuable supplement to the existing TCGA molecular classification system, providing critical support for individualized adjuvant therapy. Notably, our results indicate that despite receiving standard adjuvant treatment, most high-risk patients still had significantly poorer outcomes, suggesting the need for more intensive therapeutic strategies in this population.

Selecting the appropriate intensity of adjuvant therapy following endometrial cancer surgery remains a clinical challenge. Guided by the principles of precision medicine, the risk stratification model developed in this study offers a new approach to treatment individualization. For early-stage low-risk patients identified by the model, omitting adjuvant therapy may be considered under close surveillance, thereby avoiding overtreatment. The model provides an objective basis for safely de-escalating treatment in such cases (18, 30, 31); In contrast, standard treatment regimens may be insufficient for high-risk patients, necessitating more aggressive interventions. Specifically, high-risk patients who have not yet received standard adjuvant therapy should be encouraged to complete the full course, whereas those still classified as high-risk after standard treatment may benefit from intensified approaches. These could include upgrading radiotherapy alone to concurrent chemoradiotherapy, appropriately extending treatment duration, or exploring novel modalities such as targeted and immunotherapy. From a technical perspective, adjuvant brachytherapy effectively reduces vaginal recurrence. For patients with extensive lymphovascular space invasion or stage II disease, pelvic external beam radiotherapy (EBRT) should be administered to control pelvic and para-aortic nodal regions (32).Studies have shown that EBRT combined with vaginal brachytherapy significantly reduces locoregional recurrence risk (31). Several randomized clinical trials (including NSGO/EORTC and PORTEC-3) have further confirmed that combined chemoradiotherapy significantly improves survival outcomes in high-risk patients compared to radiotherapy alone (30, 33).

In recent years, molecular classification based on The Cancer Genome Atlas (TCGA) has significantly improved prognostic assessment in endometrial cancer, establishing four distinct molecular subtypes: POLE-mutant, p53-abnormal, mismatch repair-deficient, and no specific molecular profile (NSMP). The NSMP subtype accounts for approximately 30%–40% of cases (10) and exhibits high heterogeneity in both clinicopathological and molecular characteristics, with an intermediate prognosis. This underscores the urgent need for effective stratification tools to optimize clinical management. The predictive model developed in this study, which integrates IGF2BP2 with clinicopathological parameters, represents a significant step toward refined risk stratification for NSMP patients, providing a basis for individualized treatment adjustments. Despite the clinical value of TCGA classification, its widespread adoption faces challenges: the current system has limitations (e.g., some high-copy-number tumors lack TP53 mutations) (34, 35); and comprehensive molecular testing remains impractical in resource-limited settings. Therefore, combining novel biomarkers with traditional pathological features for comprehensive risk assessment has become an important research direction. The present nomogram can serve as a practical and cost-effective transitional tool, complementing the existing molecular classification system until comprehensive molecular profiling becomes universally accessible.

This study has several limitations. First, the model was developed using retrospective data, which may be subject to selection bias, treatment heterogeneity, and missing data. Second, although the multicenter design enhances generalizability, variations in diagnostic and treatment standards across institutions may introduce confounding. Additionally, this study only preliminarily explored the prognostic value of p53 and did not systematically integrate key molecular features such as POLE mutations or MSI status. Furthermore, the IGF2BP2 detection process requires standardization to improve reproducibility. Future research should validate the model in prospective cohorts and further investigate the therapeutic potential of targeting IGF2BP2.

In summary, this multicenter study confirms the independent prognostic value of IGF2BP2 in endometrial cancer recurrence. The nomogram that incorporates IGF2BP2 with traditional clinicopathological factors significantly improves recurrence risk prediction accuracy. While demonstrating robust statistical performance in our cohorts, the model’s translation to clinical practice requires prospective validation of its impact on patient management and outcomes.

## Data Availability

The original contributions presented in the study are included in the article/**Supplementary Material**. Further inquiries can be directed to the corresponding authors.
