# Supplementary material for: Predictive value of IGF2BP2 for endometrial cancer recurrence: a multicenter study
Source: Front Oncol. 2026 Feb 6;16:1733447. doi: 10.3389/fonc.2026.1733447 (PMC12920223; doi:10.3389/fonc.2026.1733447)
Supplement: Supplementary file 1 [file DataSheet1.pdf]

Supplementary Attachment 1.Association of IGF2BP2 expression levels with p53, ER, and PR status

Group	IGF2BP2 low-expression, N = 372	%	IGF2BP2 high-expression,N = 173	%	p value
**P53**					0.001
Normal	259	69.6	94	54.3	
Abnormal	113	30.4	79	45.7	
**ER**					<0.001
negative	53	14.2	48	27.7	
positive	319	85.8	125	72.3	
**PR**					<0.001
negative	64	17.2	58	33.5	
positive	308	82.8	115	66.5	

Abbreviations: IGF2BP2, insulin-like growth factor 2 mRNA-binding protein 2; ER, estrogen receptor; PR, progesterone receptor.
